# Sanitary Relations of Texas and Mexico

**Published:** 1892-12

**Authors:** 


### SANITARY RELATIONS OF TEXAS AND MEXICO.

THE AMERICAN PUBLIC HEALTH ASSOCIATION held the meeting so long announced, at Mexico City, on the 29th and 30th of November, and 1st and 2nd of December, inst.

Drs. Swearingen, State Health Officer of Texas, Dr. T. J. Bennett, Editor of the *Texas Sanitarian*, Dr. J. W. McLaughlin and Dr. F. S. White, Superintendent State Lunatic Asylum, were the representatives from Austin in attendance. The meeting was largely attended, and we learn, all was lovely. The delegates mentioned all testify to the extreme courtesy of the citizens of the City of the Aztecs ; they were shown every attention, and given a cordial welcome. Delegates were there from nearly every State ; and the Marine Hospital Service, the Medical Department of the United States Army and the United

States Navy were represented. Dr. Jenkins, the New York Health officer, who obtained such notoriety, and received so much unjnst "cussing" in connection with our recent cholera experience, was there, and took an active interest in the proceedings.

Our contemparary, the *Texas Sanitarian*, this month will contain a full account of the meeting and some of the papers which were read.

State Health Officer Swearingen read a paper on "The Sanitary Relations of Texas and Mexico," which, we learn, created a sensation. He talked "plain United States" to the Aztecs ; told them some plain unvarnished and very unpalatable truths about their neglect of sanitary principles ; and entered a protest in behalf of the public health of Texas against a continuation of this neglect. He asked that something be done by the Mexican government in the way of co-operation with Texas health officials towards eradicating smallpox, which disease is endemic in Mexico, and the JOURNAL is reliably informed that no effort whatever, is made for its suppression, or to prevent its spread. On the contrary, the masses, the poorer people, are in dense ignorance, and know nothing of the protective value of vaccination,—and wouldn't accept protection were it offered them. They hold very primitive views, and still follow the practices and customs of their ancestors—actually courting infection. We are told that they will travel miles purposely to expose their children to the disease. While the authorities, if they have any laws on the subject, make no effort to enforce them. This being the case there is little hope of its suppression unless the Mexican officials can be induced to institute compulsory vaccination ; or, at least to adopt the civilized plan of dealing with an outbreak, such as was so successfully practiced in Texas last winter.

In the winter of 1891–2, according to Dr. Swearingen's paper, there was smallpox in twenty-six counties in Texas. In every instance where the disease appeared it could be traced to primary introduction from Mexico. To suppress these outbreaks cost the State of Texas, Dr. Swearingen estimates, $150,000.

Under the circumstances,—our close relations,—business and social, and considering the vast extent of exposed border, unless an intelligent and hearty co-operation is at once established between the health authorities of the two countries, we may expect a repetition of this costly experience every winter indefinitely.

It is therefore gratifying to learn that our zealous State health officer succeeded in awakening an interest in the subject in the minds of our neighbors, and that it is highly probable that such means will be instituted as will eradicate the pest from the soil where it has taken such firm root and flourishes perennially.

The debate which followed the reading of Dr. Swearingen's paper was animated and energetic, and resulted in a resolution by Dr. Guion, of the United States Navy, providing for a committee of thirteen to be selected from the United States, Canada, and Mexico, to formulate some such plan of action as was indicated in Dr. Swearingen's paper. This resolution was loaded down with resolutions and amendments till it was thought the whole subject would be swamped, but after considerable skirmishing a motion prevailed to table all substitutes and amendments, and the resolution of Dr. Guion was adopted.

In this connection it will be remembered that Dr. Swearingen last year made a strong effort to bring about a sanitary conference between Texas and Mexico for the very purpose of establishing co-operation in putting down smallpox and for mutual protection, but it fell through by inaction on the part of the health authorities at Washington,—no State being competent to enter into an international treaty; such conference as was proposed, to be made effective, and its results binding on both parties alike, must have the stamp of national authority.

